# Remarks on Suicide from Suspension

**Published:** 1839-01-12

**Authors:** Meredith Clymer


### REMARKS ON SUICIDE FROM SUSPENSION.

By Meredith Clymer, M. D.

This is one of the most delicate questions the medical jurist is called to decide upon. Until recently, writers on legal medicine have been satisfied with reiterating the signs of death from hanging, given by Alberti at the commencement of the last century, with some unimportant modifications and additions. Later investigations, conducted with more accuracy, have, however, shown such diversity in the cadaveric phenomena, as to impair materially their value. Under such circumstances, the situation of the medical practitioner is often one of extreme perplexity. My object is to offer an abstract of the present state of our knowledge on this interesting subject.

Death from suspension takes place from four different causes,—1. Apoplexy.* 2. Asphyxia. 3. Apoplexy and Asphyxia combined; sometimes one predominating, and sometimes the other. 4. Injury to the spinal column. To these Fleichmann imagines a fifth may be subjoined—compression of the nerves of the neck, causing paralysis of the heart and lungs—and an experiment of Sir Benjamin Brodie on a Guinea pig, which to me appears very unsatisfactory and inconclusive, is adduced as evidence in its favour. Professor Remer, (Annales d'Hygiéne, Vol. IV.) reports his observations on one hundred and two cases of death by suspension, of which the proximate cause was:

| | |
|---|---|
| Apoplexy in | 9 |
| Asphyxia | 6 |
| Both conditions | 68 |
| Unknown | 19 |
| | 102 |

In one hundred and six cases recorded by Dr. Caspar of Berlin, death occurred:

| | |
|---|---|
| From apoplexy in | 9 |
| Asphyxia | 14 |
| Both conditions | 62 |
| Neither | 5 |
| Unknown | 16 |
| | 106 |

The position of the noose, doubtless, influences the result. According to Fleichmann, when the cord passes immediately over or below the larynx, or over the os hyoides, or between it and the chin, in each case not involving the mastoid processes, evidences of pure apoplexy, or of apoplexy and asphyxia, will generally be discovered. Should the cord, however, embrace the mastoid processes, and the angles of the inferior maxillary bone, its position in other respects being the same as above, death will occur from suffocation, the bony prominences preventing compression of the cervical vessels.

Of twenty cases of suicide by hanging, examined by Esquirol, dislocation of the second cervical vertebra was discoververd but in one. In the two hundred and eight cases collected by Remer and Caspar, it is not mentioned once. We are hence authorized to conclude, that except under peculiar circumstances it is a very rare termination.

The following are the principal signs of death by hanging usually given by writers in juridical medicine:—A discolored, ecchymosed, and depressed circle around the neck, in whose circumference one or more patches of excoriation may be detected. Contusion and rupture of the larynx and of the first rings of the trachea, with rupture of the muscles of the os hyoides. A livid, swollen tongue, either recurved or protruding beyond the teeth which are closed upon it. Bloody mucus in the throat, nostrils, and around the mouth. Injection of the eyes, with blueness and tumefaction of the lids, which are half shut; the lips tumid and livid; rigidity of the body; contraction of the fingers, and ecchymosis of the arms and thighs. The heart, lungs, and brain, gorged with blood.* That these are, however, very variable, and far from being unequivocal evidence of death from suspension, will be seen on a more minute examination of these several phenomena.

---

* A limited signification must here be attached to the term apoplexy; it denoting merely a highly congested state of the cerebral vessels, unaccompanied by sanguineous extravasation.

* Shakespeare thus describes "Duke Humphrey's timeless death."

"But, see, his face is black, and full of blood;
His eye-balls further out than when he lived,
Staring full ghastly like a *strangled* man;
His hair upreared, his nostrils stretched with struggling;
His hands abroad displayed."

*Henry VI. Pt. II. a. 3; s. 2.*

*Face.*—The color of the countenance is connected with the immediate cause of death. In some cases it is pale and composed, with, however, a besotted expression. Death here is probably caused by the sudden exclusion of air from the lungs. So frequently indeed is there little or no alteration exhibited in the appearance of the face, that Esquirol considers tumefaction and lividity to be post-mortem phenomena, caused by the continued presence of the cord, and in one case he saw it distinctly occur eight hours after death. Fleichmann entertains the same views.

The position of the tongue varies. In some cases it is thrust beyond the mouth, and in others it reaches only to the posterior surface of the incisors. In one instance Devergie found it bent upon itself. Smith, Foderé, Belloc, and Orfila, consider the state of the tongue to be influenced by the position of the cord, and that when it is below the hyoid bone protrusion will invariably be the consequence. In thirteen cases given by Devergie, where the cord was between the os hyoides and the thyroid cartilage, in six the tongue was found behind the teeth, in four within the mouth, and in three only was it locked between the teeth. In a body where death was evidently caused by drowning, he saw protrusion of this organ; also, in two cases where the cord was applied above the hyoid bone; and he states that he has frequently produced it in the subject by the cord in the same situation. He is disposed to consider it as resulting from nervous agency. Fleichmann is inclined to attribute it to the duration of the final struggle, and when that has been prolonged, the tongue, he thinks, will be found protruded and swollen.

*The Neck.*—The impression of the cord was for some time considered as conclusive evidence of suspension having taken place during life. In cases of suicide from hanging a furrow traverses obliquely the neck from behind forwards. Its depth is proportionate to the thinness of the cord and to the weight of the suspended body. The position of the cord varies in different cases. In forty-seven cases reported by Remer it was found to be:

| | |
|---|---|
| Between the chin and larynx in | 38 |
| Over the larynx | 7 |
| Below the larynx | 2 |
| | 47 |

In thirty-six cases recorded by Devergie it was:

| | |
|---|---|
| Above the larynx in | 21 |
| Over | 7 |
| Uncertain | 8 |
| | 36 |

In one hundred and six mentioned by Caspar, it was:

| | |
|---|---|
| Between the os hyoides and larynx in | 59 |
| On the larynx or thyoid cartilage | 9 |
| Position undetermined | 38 |
| | 106 |

According to Remer, when the cord is found above the larynx, the presumption is in favour of suicide, and this opinion the above statement would seem to confirm, it occurring in one hundred and eighteen cases out of one hundred and eighty-nine.

The color of the furrow is often the same with the skin of the neck, generally with violet coloured margins from one to two lines in depth, the injection of the superior lip being commonly more distinct than that of the inferior. This is best marked in front where the furrow is deepest. When the cord is coloured, as a black handkerchief, the furrow is frequently stained by it. When this occurs it is strong evidence in favour of suspension during life, and for obvious reasons.

When the cord is new, hard, and twisted, one or more abrasions may be visible in the furrow. When these take place during life, they are bloody; should, however, these excoriations have become hardened and dry with the skin, a vascular injection will be seen on holding the portion of intigument before a light.

In most cases the skin of the furrow presents a brownish-yellow semi-corneous appearance, resembling parchment, or a blistered surface after death, first noticed and described by Esquirol. This feels and cuts like leather. In seventy-one of Caspar's cases it occurred in fifty, and in nearly the whole of those appended by Marc to his investigations into the Prince de Condé's death. Devergie believes that it only occurs under two circumstances. 1. Where much force has been used in the application of the cord. 2. Where it has been removed immediately on the extinction of life, or where it has been suffered to remain for several days. He looks on it simply as the result of cutaneous desiccation from atmospheric influence. The fluids being driven out by pressure of the ligature, the dermal laminæ are brought in apposition, and sudden evaporation of the residue moisture taking place, desiccation occurs. The blood forced out, dyes the lips and edges of the furrow of a violet hue. It has occurred in one hour after death, before removal of the cord.* This parchment condition, is always best marked over the thyroid cartilage and sterno-mastoid muscle. Marc considers it as proof positive of suspension during life, which is denied by Esquirol and Devergie, who produced it repeatedly on the subject a few hours subsequent to death; and recent experiments made by Caspar of Berlin, confirm their statements.

*The Cellular Tissue of the Neck.*—In all cases of suspected suicide from suspension, the cellular tissue of the neck should be carefully examined. On dissecting back the integument, all the subcutaneous cellular tissue being left on the muscles, a white cellular band will be perceived, presenting one of two appearances—a shining silvery aspect, when the examination takes place a few hours after death, or the body is exposed

* Brit. and For. Med. Rev., Vol. II. p. 420.

more than twenty-four, or thirty-six hours—or a dull white cellular line. These are pure mechanical effects. The cells of the tissue being condensed by the pressure of the cord, their fluid and adipose contents are forced out, and their laminæ coming in contact, the glistening appearance results in proportion to the amount of moisture in the part. This cellular band is most distinct on the thyroid cartilage, and sterno-mastoid muscle, and less frequently on the splenius and complexus.

*Ecchymosis* was for a long time considered as a constant phenomenon in suicide, from hanging. De Haën was the first who showed that it was not of universal occurrence, and subsequent investigations prove its great infrequency.

| | | |
|---|---|---|
| In 15 cases | Klein found ecchymosis in | 0 |
| 12 | Esquirol | 0 |
| 25 | Devergie | 0 |
| 6 | Fleichmann | 2 |
| — | | — |
| 58 | | 2 |

In seventy-one cases collected from authors, by Caspar, of Berlin, ecchymosis occurred in twenty-one, or in about one in three and a half; and of fifty cases, collected by Devergie, in three only.*

Dr. Duncan, jr., was the first writer who suggested the probability of ecchymosis in the furrow depending on the direction and violence of the final struggles. Dr. Beck embraces this opinion. Remer supposes where ecchymosis was absent, that the immediate cause of death is apoplexy. From this opinion Caspar dissents, having noticed its absence as frequently when the vessels of the brain were congested, as when they were empty. He relates a case where the body was cut down half an hour before life was totally extinct; when death actually occurred *deep ecchymosis* suddenly appeared in the superficial mark of the cord. Orfila, Beuadé, Caspar, &c., failed to produce ecchymosis after death.

*State of the Larynx and Os Hyoides.*—These are generally uninjured in cases of suicide, and their fracture is, according to Devergie, presumption in favor of homicide. Valsalva mentions an instance of the hyoid-laryngeal muscles, and another where the sterno-thyroid and hyo-thyroid, as well as the cricoid cartilage, were torn. Morgagni, Valsalva, and Remer, report a rupture of the larynx; Orfila, a fracture of the os hyoides.

Amussat was the first (1828) to discover a new effect from the action of the cord-division of the middle and internal coats of the carotid artery. Since, Devergie found it once on the right side in thirteen examinations. The ligature was two pack-threads of the usual thickness, and the compression was circular. In numerous experiments, made by Lenoir of the Salpetriere, immediately after death, with the finest possible ligatures, traction being made at the same time to the feet, he failed to produce it.

*The Genital Organs.*—In seventy-seven cases, Caspar met with emission of semen in nineteen, or one in three; Remer fifteen times in thirty-five cases, nearly one-half. Care must be taken not to confound the "taches spermatiques" on the linen with gonorrhœal discharges. Erection of the penis, partial or complete, is probably nearly constant. Of fourteen negroes, executed at Martinique, erection at the moment of death, according to M. Guyon, occurred in all; one hour after death, traces of it were present only in nine. The female organs are stated, by some authorities, to exhibit post-mortem evidence of turgescence. In twenty-nine cases, Caspar found it once. In a case mentioned by Esquirol, both ovaries were engorged, particularly the right.

*Other Organs.*—The condition of the lungs varies. They are sometimes found in a congested state, and at other times collapsed. Laennec's emphysema was found in the upper and middle lobes of the right lung, in a case related by Prus. The right cavities of the heart contain usually a considerable quantity of blood. The base of the tongue is frequently of a rosy tint, as is also the mucous membrane of the larynx and trachea. The hands are occasionally clenched, and the finger nails driven into the palm.

On a review of what has been said, it will be seen that the signs of suicide from suspension, are exceedingly ambiguous. The presence of certain phenomena, as ecchymosis in the course of the cord, and division of the carotid artery, may be regarded as certain evidence in its favour, but these, unhappily, are of unfrequent occurrence, and their absence is by no means conclusive, or even presumptive proof of the contrary. After, however, the practitioner becomes fully acquainted with the collateral circumstances, previous history, state of mind, &c., and if these should favour the idea of self-destruction, if the physical signs present correspond with those generally admitted, he is justified in giving an opinion to that effect; but he should bear in mind that all these may be wanting, and yet suspenion have happened during life.

### SURGICAL CASES.

Reported by WILLIAM HARRIS, M. D.

*Case of Incised Wound of the Wrist Joint,—healed by the first intention.*

J. R., a son of a respectable farmer of Chester county, aged fourteen years, whilst assisting to chop the winter's fire wood, received, accidentally, a severe and direct blow from an axe. It passed through the wrist, in a transverse direction, between the lower end of the radius and

* The result of one hundred and two cases of suicide, published by Dr. Remer, of Berlin, in which ecchymosis was present in nine-tenths, would seem at variance with the above. It should, however, be remembered that much confusion prevails as to the exact meaning of the term ecchymosis. The violet hue of the lips of the furrow is so called by some writers; others have mistaken the common cadaveric lividity for it. When it is employed in its proper signification—sanguineous extravasation into the sub-cutaneous cellular membrane—I have but little doubt that perfect accordance will be found in all the statements. It is to be remembered that Dr. Remer's cases were not the result of personal observation, but were collected from various sources, many of which might have been exceedingly inaccurate.

